# Impact of Different JAK Inhibitors and Methotrexate on Lymphocyte Proliferation and DNA Damage

**DOI:** 10.3390/jcm10071431

**Published:** 2021-04-01

**Authors:** Annika Reddig, Linda Voss, Karina Guttek, Dirk Roggenbuck, Eugen Feist, Dirk Reinhold

**Affiliations:** 1 Institute of Molecular and Clinical Immunology, Otto-Von-Guericke-University Magdeburg, 39120 Magdeburg, Germany; linda.voss@med.ovgu.de (L.V.); karina.guttek@med.ovgu.de (K.G.); dirk.reinhold@med.ovgu.de (D.R.); 2 Institute of Biotechnology, Faculty Environment and Natural Sciences, Brandenburg University of Technology Cottbus-Senftenberg, 01968 Senftenberg, Germany; dirk.roggenbuck@b-tu.de; 3 Faculty of Health Sciences, Joint Faculty of the Brandenburg University of Technology Cottbus-Senftenberg, the Brandenburg Medical School Theodor Fontane and the University of Potsdam, 01968 Senftenberg, Germany; 4 Helios-Department of Rheumatology, Cooperation Partner of the Otto-Von-Guericke-University, 39245 Vogelsang-Gommern, Germany; eugen.feist@helios-gesundheit.de

**Keywords:** JAK inhibitor, proliferation, DNA damage repair, γH2AX, PBMCs, T lymphocytes

## Abstract

Janus kinase inhibitors (JAKis) represent a new strategy in rheumatoid arthritis (RA) therapy. Still, data directly comparing different JAKis are rare. In the present in vitro study, we investigated the immunomodulatory potential of four JAKis (tofacitinib, baricitinib, upadacitinib, and filgotinib) currently approved for RA treatment by the European Medicines Agency. Increasing concentrations of JAKi or methotrexate, conventionally used in RA therapy, were either added to freshly mitogen-stimulated or preactivated peripheral blood mononuclear cells (PBMC), isolated from healthy volunteers. A comparable, dose-dependent inhibition of lymphocyte proliferation was observed in samples treated with tofacitinib, baricitinib, and upadacitinib, while dosage of filgotinib had to be two orders of magnitude higher. In contrast, antiproliferative effects were strongly attenuated when JAKi were added to preactivated PBMCs. High dosage of upadacitinib and filgotinib also affected cell viability. Further, analyses of DNA double-strand break markers γH2AX and 53BP1 indicated an enhanced level of DNA damage in cells incubated with high concentrations of filgotinib and a dose-dependent reduction in clearance of radiation-induced γH2AX foci in the presence of tofacitinib or baricitinib. Thereby, our study demonstrated a broad comparability of immunomodulatory effects induced by different JAKi and provided first indications, that (pan)JAKi may impair DNA damage repair in irradiated PBMCs.

## 1. Introduction

Rheumatoid arthritis (RA) is a chronic, autoimmune disease, characterized by inflammation and progressive damage of synovial joints, when treated insufficiently [1]. With increasing knowledge about disease pathophysiology, new pharmaceutical strategies and compounds are available. After the application of conventional synthetic disease-modifying antirheumatic drugs (csDMARDs), such as methotrexate (MTX) in late 1980s, and biological DMARDs since late 1990s, small-molecule Janus kinase inhibitors (JAKis), classified as targeted synthetic DMARDs, represent a new milestone in RA treatment [1,2,3]. Clinical studies with JAKi demonstrated similar efficacy and safety compared to biological DMARDs [4,5]. However, long-term data for JAKi covering several years are still missing.

Janus kinases (JAKs) are cytoplasmic tyrosine kinases comprising four different types of JAK enzymes in humans: JAK1, JAK2, JAK3, and tyrosine kinase 2 (TYK2) [6]. While JAK1, JAK2, and TYK2 are expressed ubiquitously, JAK3 is predominantly detectable in hematopoietic tissue [7,8]. Upon extracellular ligand binding, JAKs associate as homo- or heterodimers with type I and type II cytokine receptors. Subsequently, JAK dimers become activated by auto- and transphosphorylation and phosphorylate the cytoplasmic tail of the cytokine receptor [6,8]. This induces the recruitment and binding of signaling molecules, such as the members of the signal transducer and activator of transcription (STAT) family (seven members: STAT1/2/3/4/5A/5B/6). After JAK-mediated STAT phosphorylation, dimerization, and activation, STAT dimers translocate to the nucleus where they act as transcription factors for multiple target genes, modulating, i. a., survival, proliferation, or differentiation of T lymphocytes [9,10]. More than 50 different cytokines and growth factors are known ligands of type I/II cytokine receptors. Depending on the cytoplasmic chains of the receptor, they are able to associate either with only one type of JAK enzyme or with different JAK isoforms. Hence, this creates a high degree of specificity regarding different JAK and STAT combinations [9,10,11].

Sufficient JAK-STAT signaling is essential in the regulation of immunological processes. Polymorphisms and loss- or gain-of-function mutations within this pathway are associated with immunodeficiency, autoimmune disease, and hematological malignancy [9,12]. Therefore, JAK-targeting agents represent a new class of immunomodulatory drugs [11]. After first approval of JAK inhibiting drugs for the treatment of neoplastic diseases, several JAKi are also authorized for the treatment of RA by the U.S. Food and Drug Administration (FDA) and European Medicines Agency (EMA) [13,14]. The two approved first-generation JAK inhibitors tofacitinib (JAK3 > JAK1 > JAK2) and baricitinib (JAK1 > JAK2 > JAK3) are classified as pan-JAK inhibitors, targeting multiple JAK isoforms, but with different affinities. In contrast, the two second-generation JAKi upadacitinib and filgotinib (not yet approved by the FDA) show high selectivity for JAK1 and primarily inhibit its associated cytokine-receptors [10,15]. However, selectivity of JAKi towards specific JAK isoforms is not absolute and depends on dosage and cell type [16]. An overview of reported mean half-maximal inhibitory concentrations (IC_50_) obtained by enzymatic assays is provided in Table S1.

Although already approved for RA treatment, in vivo and in vitro head-to-head studies of all four JAKi are rare. Therefore, we evaluated the immunomodulatory and cytotoxic potential of tofacitinib, baricitinib, upadacitinib, and filgotinib on human PBMCs freshly isolated from healthy donors. For comparison with conventional synthetic DMARDs, samples treated with MTX were investigated in parallel. JAKi or MTX were either added directly to freshly PHA-stimulated PBMCs or 48 h after PBMC activation, to investigate their impact on preactivated T lymphocytes, as this might be more relevant regarding inflammatory conditions in vivo [17]. Compared to healthy controls, peripheral blood isolated from patients with active RA revealed an enhanced level of activated PBMCs, which may play a direct role in disease pathogenesis [18]. Furthermore, Kitanaga et al. stated constitutive activation of JAK/STAT signaling in PBMCs from patients with systemic sclerosis or RA [19].

JAK/STAT signaling is involved in regulation of multiple fundamental cellular processes. Additionally, there is increasing evidence suggesting that JAK/STAT signaling also modulates molecules involved in DNA damage response pathways [20,21,22,23,24]. To investigate the impact of JAKi on DNA double-strand break (DSB) formation and on repair of radiation-induced DNA damage we quantified nuclear foci stained by γH2AX or 53BP1 (p53-binding protein 1) antibodies. These markers have been described as sensitive molecular indicators for DNA DSBs [25,26,27].

The objective of the present in vitro study was to compare the immunomodulatory potential of all four JAKi currently approved for RA treatment in Europe. Therefore, we treated freshly and preactivated PBMCs with rising concentrations of either tofacitinib, baricitinib, upadacitinib, filgotinib, and MTX and determined the effect on cell proliferation, activation (CD25) and apoptosis. Furthermore, we investigated the impact of different JAKi and MTX on DNA damage induction and repair by fluorescence microscopic analysis of DNA DSB markers γH2AX and 53BP1. Our study indicates a broad comparability of the immunomodulatory effects induced by different JAKi and offers a first indication, that (pan)JAKi may impair DNA damage repair in radiated lymphocytes.

## 2. Experimental Section

### 2.1. Ethics Statement

The study was performed in accordance with the Declaration of Helsinki and was approved by the local ethics committee (No. 183/20). All 14 healthy blood donors (10 female and 4 male; mean age: 35 ± 12 years) who agreed to participate in this study provided written informed consent.

### 2.2. Cell Culture

Human peripheral blood mononuclear cells (PBMCs) were isolated from heparinized blood by density gradient centrifugation using Pancoll separating solution (PAN-Biotech, Aidenbach, Germany). Afterwards, PBMCs were washed twice and suspended to a final density of 1 × 10^6^ PBMCs per mL in serum-free AIM-V culture medium (Invitrogen, Eggenstein, Germany). Activation of T lymphocytes among PBMCs was achieved by mitogen stimulation with 2 µg/mL phytohemagglutinin (PHA, life technologies/Gibco, London, UK).

JAK inhibitors tofacitinib, baricitinib, upadacitinib, and filgotinib and methotrexate (MTX) were all purchased from Selleckchem (Houston, TX, USA). These agents (stock solution: 10 mM in dimethyl sulfoxide (DMSO)) were either added simultaneously with PHA into cell culture plates or 48 h after PHA-stimulation, to investigate their impact on preactivated PBMCs. Cells treated with DMSO, diluted 1:1000, served as corresponding vehicle controls. 

### 2.3. Proliferation Analysis by ^3^H-Thymidine Incorporation

Cell proliferation was analyzed by ^3^H-thymidine incorporation assay. For T cell activation, PHA was added to PBMC suspension and 1 × 10^5^ PBMCs/well were seeded into flat bottom 96-well plates. Different concentration (1 nM–10 µM, 1:10 serial dilution) of the four investigated JAKi were added as triplicates either directly to the cell culture or 48 h after PHA-stimulation (preactivated lymphocytes). After 72 h of PHA activation, PBMCs were pulsed with [^3^H]-thymidine at a dose of 0.2 μCi/well for additional 6 h. At the end of the incubation period cells were harvested and ^3^H-thymidine incorporation was quantified using the microplate liquid scintillation counter Wallac MicroBeta TriLux from Perkin Elmer (Waltham, MA, USA).

### 2.4. Proliferation Analysis by the CFSE Dilution Assay

Additionally, cell proliferation was assessed by the cell trace carboxyfluorescein succinimidyl ester (CFSE) cell proliferation kit (Invitrogen, Carlsbad, CA, USA). Therefore, freshly isolated PBMCs were washed in phosphate buffers saline (PBS; PAN-Biotech) and resuspended in 1 mL PBS containing 5% inactivated fetal calf serum (FCS). Subsequently, 5 µM CFSE solution was added and incubated for 5 min at room temperature in the dark. Afterwards, cells were washed twice in PBS-FCS and were resuspended to a final concentration of 1 × 10^6^ PBMC/mL in AIM-V medium. CFSE loaded unstimulated cells served as control sample, representing CFSE^high^, non-divided cell population. For activation PHA was added to remaining CFSE-stained PBMC suspension, which was subsequently plated into 24-well cell culture plates. Increasing concentrations of JAKi or MTX were either added directly or 48 h after PHA-stimulation. After an incubation period of 96 h PBMCs were transferred into 5 mL round bottom polystyrene tubes and washed once in cold PBS containing 0.5% BSA. Subsequently, CFSE intensity of living cells (gating based on forward/side scatter signal) was determined in FITC channel by flow cytometry (LSRFortessa cell analyzer, BD Biosciences, Mountain View, CA, USA) and FlowJo software (version 7.6.4, Tree Star Inc., Ashland, OR, USA).

### 2.5. Analysis of CD25 Expression

Activation status was assessed by CD25 expression. Therefore, 1 × 10^6^ PBMCs/sample were simultaneously treated for 48 h with PHA and different JAKi at various concentrations as indicated. Afterwards, cells were transferred into 5 mL round bottom polystyrene tubes, washed twice with PBS containing 0.5% bovine serum albumin (BSA; AppliChem, Darmstadt, Germany) and incubated for 30 min at 4 °C with 1:200 diluted phycoerythrin(PE)-coupled anti-human CD25 antibody (BioLegend, San Diego, CA, USA). Subsequently, samples were washed with PBS containing 2 mM ethylenediaminetetraacetic acid (EDTA) and resuspended in PBS-BSA. Samples were kept cold until flow cytometry analysis using a BD LSRFortessa cell analyzer. Data were acquired by FACSDiva 6.0 software (BD Biosciences) and analyzed by FlowJo software. 

### 2.6. Viability Assessment

JAKi- and MTX- induced cell death was determined after 72 h in unstimulated, freshly PHA-stimulated and preactivated PBMCs using a FITC Annexin V/propidium iodide (PI) apoptosis detection kit (BioLegend, San Diego, CA, USA). In brief, 1 × 10^6^ PBMC were transferred into 5 mL round bottom polystyrene tubes, washed once in cold PBS-BSA and resuspended in 50 µL staining solution, comprising Annexin V and PI diluted 1:20 in Annexin V binding buffer. After 15 min incubation at room temperature in the dark cellular staining was terminated by addition of 200 µL binding buffer. Ratio of viable (Annexin V^−^/PI^−^), early apoptotic (Annexin V^+^/PI^−^), late apoptotic (Annexin V^+^/PI^+^), and necrotic (Annexin V^−^/PI^+^) cells was determined of 20,000 cells/sample by BD LSRFortessa cell analyzer and subsequent analysis utilizing FlowJo software. Unstained, single- and double-stained cells treated with camptothecin were included as control samples.

### 2.7. Detection of γH2AX and 53BP1 Foci

Automated quantification of intranuclear γH2AX and 53BP1 foci, described as sensitive indicators for DNA double-strand breaks (DSB), was performed to study JAKi-induced DNA DSB and their impact on DNA repair of radiation-induced DSBs [25,26,27]. Therefore, unstimulated PBMCs were treated with indicated concentrations of JAKi or MTX. Additionally, one fraction was exposed to γ-rays at a dose of 2 Gy (Biobeam 8000, Cs 137, Gamma-Service Medical, Leipzig, Germany) to induce DNA damage. After an incubation period of 24 h non-irradiated and radiated samples were harvested, washed in PBS and fixed for 15 min with 1% formaldehyde on silanized glass slides, as described in detail elsewhere [28,29]. Subsequently, cells were permeabilized in 0.2% Triton X-100, washed in blocking buffer (PBS containing 1% BSA) and incubated for 60 min at room temperature simultaneously with 1:1000 diluted γH2AX (anti-phosphohistone H2AX mouse monoclonal IgG primary antibody, clone JBW301, Millipore, Schwalbach, Germany) and 53BP1 primary antibodies (anti-53BP1 rabbit polyclonal IgG (NB 100–305), Novus Biologicals, Centennial, CO, USA). Afterwards, slides were washed and incubated for 1 h at room temperature with 1:500 diluted polyclonal goat anti-mouse IgG antibody conjugated to Alexa Fluor 488 and polyclonal goat anti-rabbit IgG antibody conjugated to Alexa Fluor 647 (Lifetechnologies, Darmstadt, Germany). After a final washing cycle in PBS, slides were covered with DAPI (4′,6-diamidino-2-phenylindole)-containing mounting medium (Medipan, Berlin/Dahlewitz, Germany). Directly after staining procedure slides were analyzed by an automated digital microscopy system (AKLIDES, Medipan, Berlin/Dahlewitz, Germany) quantifying the number of γH2AX and 53BP1 foci in 300 nuclei per sample [28,29]. 

### 2.8. Statistical Analysis

Quantitative data analysis was performed by GraphPad Prism software version 5.01 (Graph Pad Software, La Jolla, CA, USA). Half-maximal inhibitory dose (IC_50_) was calculated by non-linear regression from logarithm-transformed data. Significance levels among samples treated with the same JAKi or MTX were calculated by repeated measures ANOVA (analysis of variance) with 95% confidence interval (α = 0.05) followed by the Dunnett’s post-hoc test, to compare the results with DMSO-treated control group. Data in text and figures are displayed as the mean ± standard error of the mean (SEM), and *p* values are indicated by asterisks (*** *p* ≤ 0.001; ** *p* ≤ 0.01; * *p* ≤ 0.05).

## 3. Results

### 3.1. Impact of JAKi and MTX on Lymphocyte Activation and Proliferation

#### 3.1.1. ^3^H-Thymidine Incorporation

**Figure 1:**
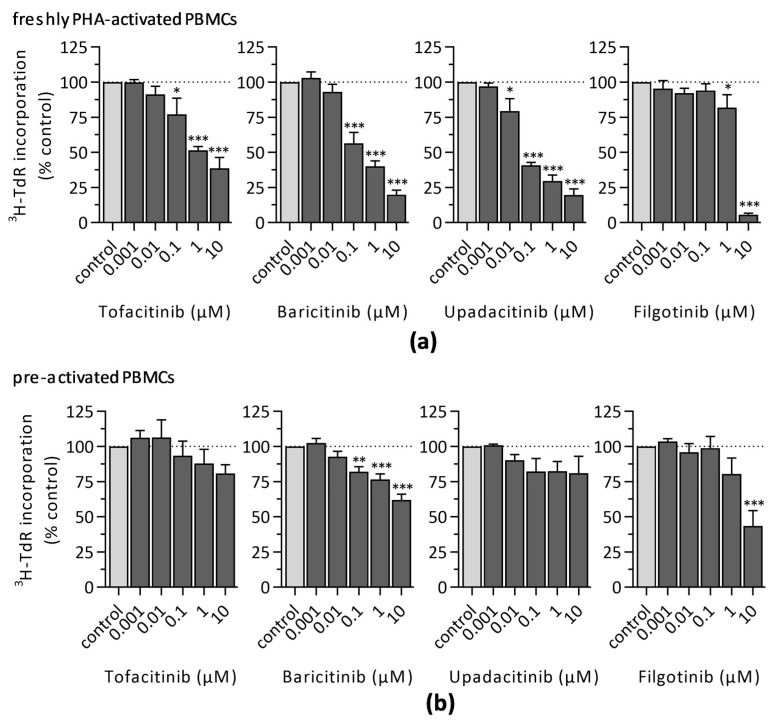
Proliferation analysis by ^3^H-thymidine incorporation assay 72 h after PHA-stimulation of PBMCs treated with indicated concentrations of JAKi either (**a**) immediately after activation or (**b**) 48 h after PHA-stimulation. Diagrams display the mean ± SEM of five independent experiments normalized to the DMSO control (*** *p* ≤ 0.001; ** *p* ≤ 0.01; * *p* ≤ 0.05).

To assess the impact of different JAKi on lymphocyte proliferation DNA synthesis was analyzed by ^3^H-thymidine incorporation 72 h after PHA-stimulation (Figure 1). As expected, lymphocyte proliferation was significantly inhibited in freshly stimulated PBMCs by all four investigated JAKi in a dose-dependent manner (Figure 1a). However, whereas tofacitinib, baricitinib, and upadacitinib showed significantly inhibitory effects already at the nanomolar dose range, higher concentrations of filgotinib were required to reduce lymphocyte proliferation to a similar extent. Though, treatment of PBMCs with 10 µM filgotinib showed the strongest decrease in ^3^H-thymidine incorporation (5.5% ± 1.0%) when compared to cell cultures treated with 10 µM tofacitinib (38.6% ± 7.6%), baricitinib (19.8% ± 3.3%), or upadacitinib (19.4% ± 4.6%).

Additionally, we investigated the immunomodulatory potential of these four JAKi on preactivated PBMCs. Therefore, increasing concentrations of JAKi were added to the cell culture 48 h after PHA-stimulation (Figure 1b). Assessment of ^3^H-thymidine incorporation revealed no significant inhibitory effects in preactivated lymphocytes treated with tofacitinib or upadacitinib, whereas baricitinib decreased cell proliferation in a dose-dependent manner. Filgotinib significantly reduced the level of incorporated ^3^H-thymidine only when the highest dose (10 µM) was applied. Of note, inhibitory potential of JAKi was strongly attenuated in preactivated cell cultures compared to freshly stimulated PBMCs.

However, due to assay limitations, data obtained from PBMCs treated with MTX had to be excluded from this proliferation analysis. MTX led to a concentration dependent increase of ^3^H-thymidine incorporation (Figure S1), as also observed by others [30]. This effect is caused by MTX-induced blockage of internal thymidine biosynthesis. The lack of available endogenous thymidine was overcome by enhanced incorporation of external radiolabeled thymidine provided in the cell culture medium, as reflected by an increase in counted radioactivity.

To confirm results obtained by ^3^H-thymidine incorporation and to include samples treated with MTX, proliferation was additionally analyzed by the CFSE dilution assay.

#### 3.1.2. CFSE Dilution Assay

**Figure 2:**
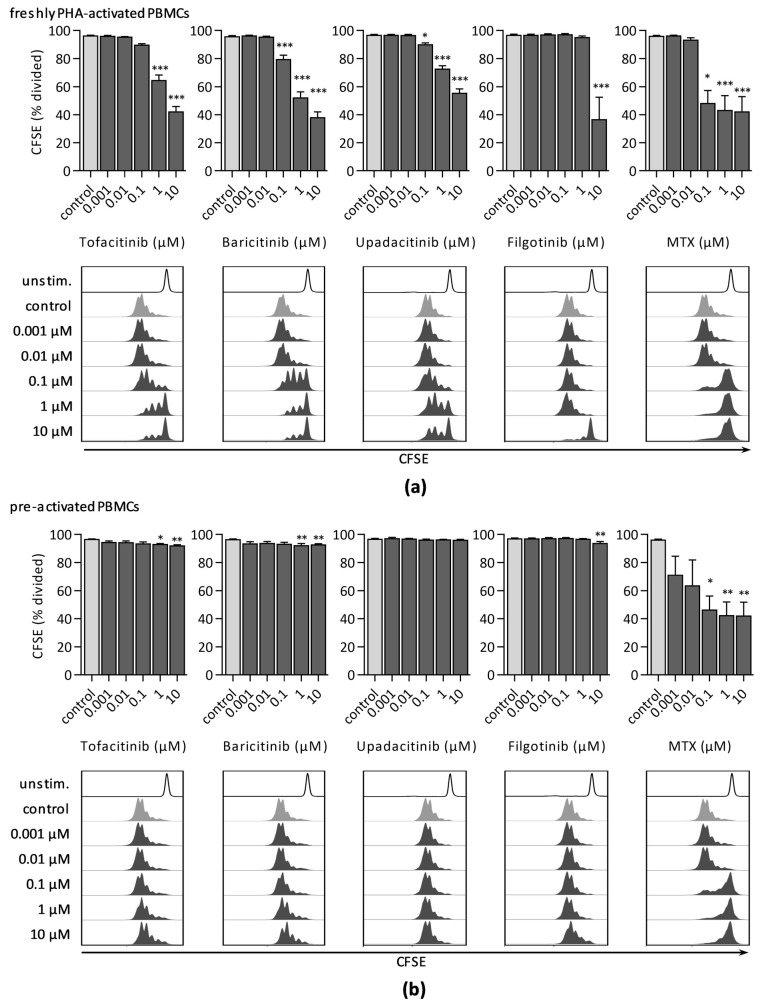
Proliferation analysis by CFSE dilution assay 96 h after PHA-stimulation of PBMCs treated with indicated concentrations of JAKi or MTX either (**a**) immediately after activation or (**b**) 48 h after PHA-stimulation. Percentage of divided cell population was quantified. Diagrams display the mean ± SEM of three independent experiments (*** *p* ≤ 0.001; ** *p* ≤ 0.01; * *p* ≤ 0.05). Representative histograms of CFSE intensity are shown below.

In line with our data obtained by ^3^H-thymidine incorporation, CFSE dilution analysis demonstrated a dose-dependent decrease in lymphocyte proliferation, when JAKi were added to freshly stimulated lymphocytes (Figure 2a). Filgotinib significantly reduced the percentage of dividing cells only when PBMCs were treated with 10 µM filgotinib (36.8% ± 15.7%), yet representing the strongest effect compared to samples cultured with 10 µM tofacitinib (42.4% ± 3.4%), baricitinib (38.3% ± 3.7%), or upadacitinib (55.5% ± 2.9%), respectively. Treatment with MTX ≥ 0.1 µM also significantly reduced cell proliferation by more than 50%.

In preactivated cell cultures, the antiproliferative impact of JAKi was reduced. Still, JAKi significantly diminished the fraction of dividing cells after treatment with either 1 µM (93.3% ± 0.3%) or 10 µM (92.4% ± 0.4%) tofacitinib, 1 µM (92.5% ± 1.0%) or 10 µM (92.9% ± 0.4%) baricitinib, and 10 µM filgotinib (94.0% ± 1.0%) (Figure 2b). In contrast, MTX strongly reduced ratio of dividing cells (<50%) at a dose range ≥ 0.1 µM when added to freshly and to preactivated PBMCs.

#### 3.1.3. CD25 Expression

**Figure 3:**
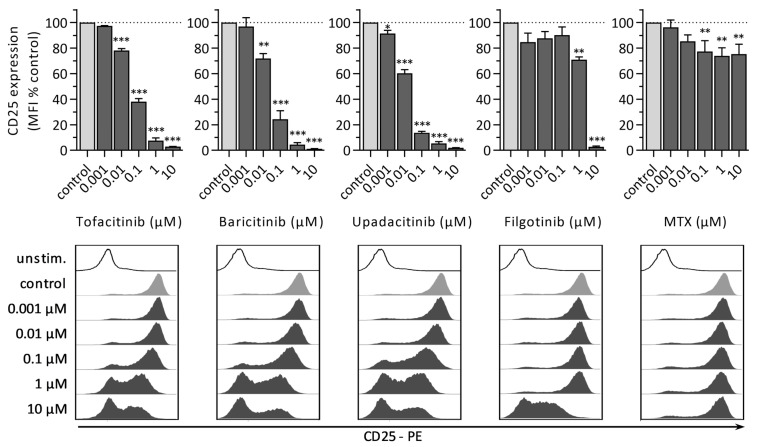
Assessment of lymphocyte activation by CD25 expression 48 h after PHA-stimulation and combined treatment of indicated JAKi or MTX concentrations. Median fluorescence intensity of CD25 expression was quantified. Diagrams display the mean ± SEM of three independent experiments (*** *p* ≤ 0.001; ** *p* ≤ 0.01; * *p* ≤ 0.05). Representative histograms of CD25 intensity are shown below.

Lymphocyte activation was assessed 48 h after simultaneous incubation of PBMCs with PHA and JAKi by the flow cytometric measurement of CD25 expression (Figure 3). Similar to proliferation analyses, JAKi and MTX induced a dose-dependent reduction of CD25 expression. Since data from CFSE analysis were insufficient to calculate half maximal inhibitory concentration (IC_50_) for all JAKi investigated, we compared IC_50_ based on the CD25 expression level. Upadacitinib (IC_50_: 0.0149 µM) showed the strongest inhibitory effect, followed by baricitinib (IC_50_: 0.0284 µM), tofacitinib (IC_50_: 0.0522 µM), and filgotinib (IC_50_: 2.4378 µM). Due to an attenuated effect of MTX on CD25 expression respective IC_50_ value could not be calculated. Nevertheless, a significant reduction of CD25 was observed for PBMCs treated with 0.1 µM (77.3% ± 8.6%), 1 µM (73.9% ± 6.4%), or 10 µM (75.3% ± 7.8%) MTX.

### 3.2. Impact of JAKi and MTX on Lymphocyte Viability

**Figure 4:**
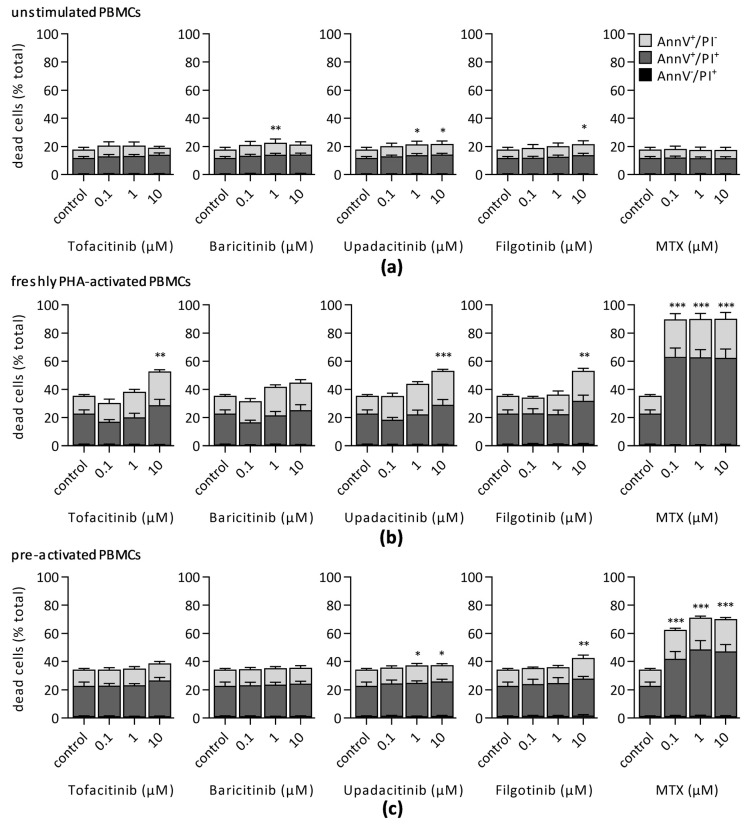
Cell viability was assessed after 72 h by Annexin V/PI staining of (**a**) unstimulated, (**b**) freshly PHA-stimulated, or (**c**) preactivated PBMCs treatment with indicated concentrations of JAKi or MTX. Percentage of Annexin V^+^/PI^−^ (light grey), Annexin V^+^/PI^+^ (dark grey), and Annexin V^−^/PI^+^ (black; <2%) population were quantified. Diagrams display the mean ± SEM of five independent experiments (*** *p* ≤ 0.001; ** *p* ≤ 0.01; * *p* ≤ 0.05).

Further, we investigated JAKi-induced cytotoxicity in unstimulated (Figure 4a), freshly stimulated (Figure 4b), and preactivated (Figure 4c) PBMCs using Annexin V/PI staining. Statistical analysis revealed a small but significant rise of apoptotic cell fraction already in unstimulated PBMCs (control: 17.9% ± 0.9%) treated either with 1 µM baricitinib (22.7% ± 2.7%), 1 µM (21.5% ± 2.3%), or 10 µM upadacitinib (21.8% ± 2.1%) or after incubation with 10 µM filgotinib (21.7% ± 2.3%) (Figure 4a). Tofacitinib and MTX did not affect viability in unstimulated PBMCs.

PHA-activation itself significantly increased the apoptotic cell population (control: 35.5% ± 2.2%) (Figure 4b). Apoptotic fraction further rose 72 h after treatment with 10 µM tofacitinib (52.8% ± 4.9%), 10 µM upadacitinib (53.2% ± 4.1%), or 10 µM filgotinib (53.2% ± 3.3%). In contrast to JAKi, simultaneous incubation of PBMCs with PHA and 0.1 µM MTX already induced a high proportion of apoptotic cells (89.7% ± 2.1%), which did not further grow with increasing MTX concentrations.

Addition of tofacitinib or baricitinib to preactivated PBMCs did not affect cell viability. However, high doses of JAK1-selective JAKi upadacitinib (1 µM: 37.2% ± 2.5%; 10 µM: 37.32% ± 2.3%) or filgotinib (10 µM: 42.5% ± 2.7%) induced a significant rise in apoptotic cell fraction. Treatment of preactivated PBMCs with MTX also strongly induced cell death (0.1 µM: 62.5% ± 5.4%).

### 3.3. Impact of JAKi and MTX on DNA Double-Strand Break Formation and DNA Repair

**Figure 5:**
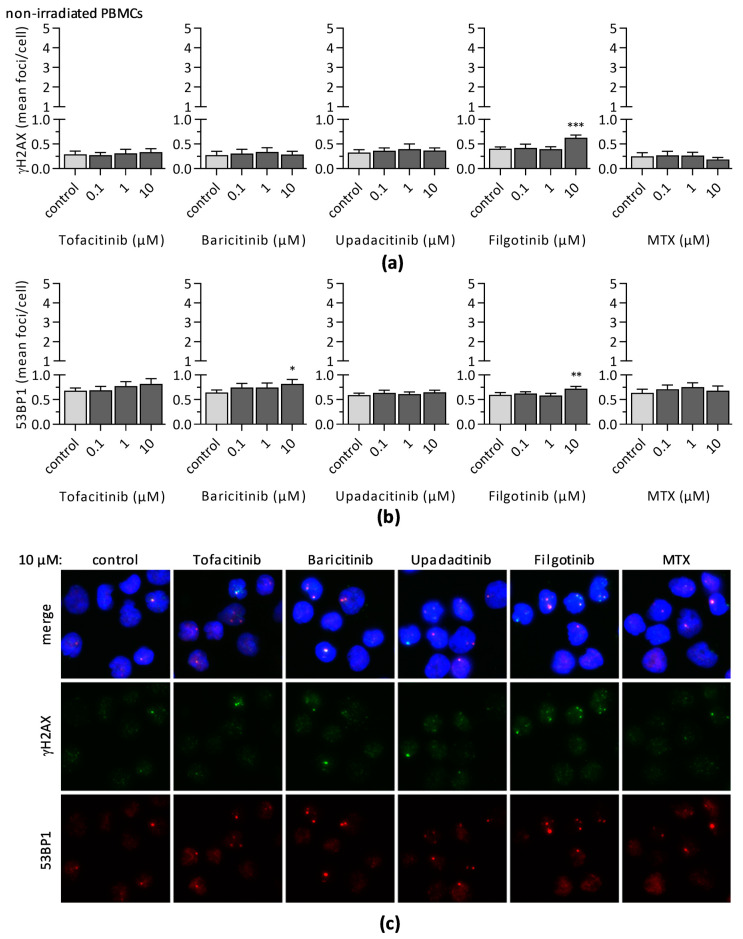
The mean number of (**a**) γH2AX and (**b**) 53BP1 foci per cell were assessed, as markers for induced DNA double-strand breaks, 24 h after treatment of unstimulated PBMCs with indicated JAKi or MTX concentration. Diagrams display the mean ± SEM of seven independent experiments (*** *p* ≤ 0.001; ** *p* ≤ 0.01; * *p* ≤ 0.05). (**c**) Representative immunofluorescence microscopy images of PBMCs from one donor treated with either 10 µM JAKi or MTX. Colors indicate DNA/DAPI (blue), γH2AX (green), and 53BP1 (red).

To analyze the impact of JAKi on DNA DSB formation we investigated the induction of γH2AX and 53BP1 foci 24 h after drug treatment (Figure 5). In general, incubation of unstimulated PBMCs with JAKi or MTX did not induce γH2AX foci, only samples treated with 10 µM filgotinib revealed a significant increase in γH2AX foci formation (control: 0.41 ± 0.04 foci/cell; 10 µM filgotinib: 0.63 ± 0.06 foci/cell). This enhanced level of DNA DSB marker after 10 µM filgotinib treatment was further confirmed by a significant rise of 53BP1 foci (control: 0.59 ± 0.05 foci/cell; 10 µM filgotinib: 0.72 ± 0.04 foci/cell). Additionally, an increase of 53BP1 foci was also observed in cells treated with 10 µM baricitinib (0.82 ± 0.09 foci/cell).

**Figure 6:**
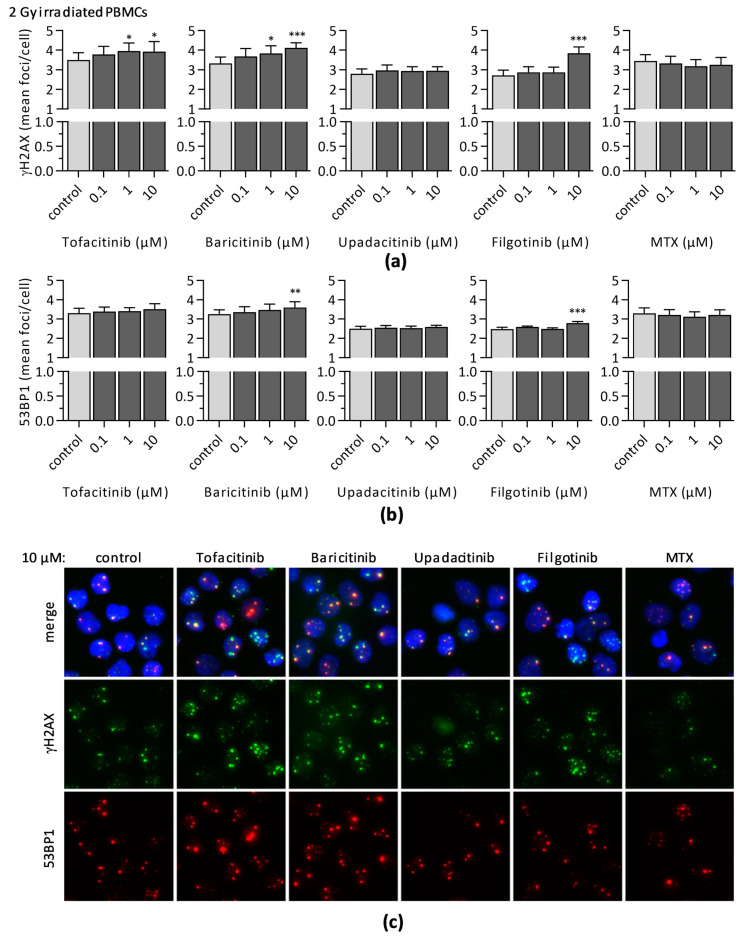
The mean number of residual (**a**) γH2AX and (**b**) 53BP1 foci per cell were assessed 24 h after 2 Gy irradiation of unstimulated PBMCs in the presence of indicated JAKi or MTX to analyze DNA repair by means for foci clearance. Diagrams display the mean ± SEM of seven independent experiments (*** *p* ≤ 0.001; ** *p* ≤ 0.01; * *p* ≤ 0.05). (**c**) Representative immunofluorescence microscopy images of PBMCs from one donor treated with either 10 µM JAKi or MTX. Colors indicate DNA/DAPI (blue), γH2AX (green), and 53BP1 (red).

Assessment of residual γH2AX and 53BP1 foci 24 h after radiation was applied to analyze DNA repair efficacy by means of foci clearance. Therefore, JAKi or MTX were added to cell cultures, which were subsequently irradiated with 2 Gy γ-radiation. Remaining foci were determined 24 h after irradiation (Figure 6). A significant, dose-dependent enrichment of residual γH2AX was determined in samples treated with pan-JAKi tofacitinib (control: 3.50 ± 0.37 foci/cell; 1 µM tofacitinib: 3.96 ± 0.41 foci/cell; 10 µM tofacitinib: 3.93 ± 0.51 foci/cell) or baricitinib (control: 3.33 ± 0.32 foci/cell; 1 µM baricitinib: 3.84 ± 0.39 foci/cell; 10 µM baricitinib: 4.12 ± 0.29 foci/cell). Radiated PBMCs incubated in the presence of 10 µM baricitinib also revealed a significant increase in the 53BP1 foci level (control: 3.25 ± 0.23 foci/cell; 10 µM baricitinib: 3.60 ± 0.29 foci/cell). Furthermore, treatment of irradiated PBMCs with 10 µM filgotinib correlated with enhanced level of residual γH2AX foci (control: 2.71 ± 0.27 foci/cell; 10 µM filgotinib: 3.85 ± 0.32 foci/cell) and 53BP1 foci (control: 2.48 ± 0.10 foci/cell; 10 µM filgotinib: 2.79 ± 0.09 foci/cell). Of note, differences in foci levels of DMSO controls among groups were caused by individual variance among blood donors, since some additional experiments investigating the impact of upadacitinib and filgotinib had to be performed separately.

## 4. Discussion

JAK inhibitors represent a new class of immunomodulatory drugs, currently being approved for therapy of cancer and inflammatory diseases [10,11]. In comparison to biological DMARDs, which are expensive to manufacture, require cold storage, and have to be administered parenterally, small molecular JAKi circumvent these limitations. Furthermore, JAKi are compounds that can be taken orally and typically exhibit a dose-proportional pharmacokinetic profile and a short half-life in the range of hours [4,31]. This allows rapid reversal of immunosuppressive or potential drug-induced adverse effects [4]. Although not just targeting one specific cytokine, but rather signaling pathways of multiple cytokine receptors, JAKi show similar efficacy and safety profiles compared to biological DMARDs, like TNF inhibitors [4,5,13]. However, analysis of currently available data revealed, e.g., an increased frequency of herpes zoster infection and thromboembolic adverse events in RA patients receiving JAKi, while the incidence of respiratory or urinary tract infections and recorded malignancies were similar compared to other DMARDs [4,9,13]. Completion of ongoing long-term extension studies and increasing prescription rates of JAKi will provide more pharmacovigilance data also concerning potential long-term effects [32].

Currently, leading pharmaceutical authorities, such as the FDA (United States), the EMA (Europe), and the Pharmaceuticals and Medical Devices Agency (PMDA) in Japan licensed the three JAKi tofacitinib (Xeljanz^®^), baricitinib (Olumiant^®^), and upadacitinib (Rinvoq^®^) for the treatment of patients with active RA, who responded inadequately to conventional therapies [13,14]. In September 2020 a fourth JAKi, filgotinib (Jyseleca^®^), received approval by the EMA and PMDA. In contrast, the FDA has rejected filgotinib for RA treatment, raising concerns, e.g., regarding its impact on sperm parameters and the risk–benefit profile of 200 mg dose [14,33]. In Asian countries, such as Japan and Korea, an additional pan-JAKi, peficitinib (Smyraf^®^), has also been approved for the treatment of patients with moderate RA [34,35].

Due to the absence of head-to-head trials, direct comparison of JAKi with respect to their efficacy in RA treatment is limited [36,37]. In the present in vitro study, we investigated all four JAKi currently approved by the EMA for RA treatment to gain more information about their immunomodulatory potential. In contrast to previous studies, which analyzed different JAKi and their inhibitory profile concerning specific cytokine receptor signaling pathways, we determined their inhibitory potential with regard to overall lymphocyte proliferation and activation [17,38].

Therefore, PBMCs were isolated from healthy volunteers and T cells contained therein were activated by addition of PHA. Increasing concentrations of tofacitinib, baricitinib, upadacitinib, filgotinib, or MTX were added to cell cultures either directly or 48 h post PHA-stimulation, to analyze the impact on preactivated lymphocytes. Proliferation and activation were assessed by ^3^H-thymidine incorporation analysis, CFSE dilution assay, and CD25 expression. Although small numerical differences have been observed, freshly activated lymphocytes incubated with tofacitinib, baricitinib, or upadacitinib exhibited a comparable, dose-dependent inhibition of T lymphocyte proliferation and CD25 expression. In contrast, concentrations of filgotinib had to be approximately two orders of magnitude higher to induce significant inhibitory effects. This deviation also reflects the higher dosage of filgotinib regarding IC_50_ values of JAK1-enzymatic inhibition and administered concentrations in clinical studies [16,38].

As JAKi are also administered under inflammatory conditions, it is of interest to investigate their immunomodulatory potential on previously activated lymphocytes [17]. Therefore, different JAKi were added to cell cultures 48 h after PHA-stimulation. Compared to direct JAKi exposure, inhibitory effects of JAKi on lymphocyte proliferation of preactivated cells were strongly attenuated. While the CFSE assay revealed a small but significant dose-dependent decrease in cultures treated with high doses of tofacitinib, baricitinib, or filgotinib, no alterations were observed in preactivated samples incubated with upadacitinib. Data obtained from ^3^H-thymidine incorporation only revealed a significant, dose-dependent reduction of DNA synthesis in preactivated cells subsequently incubated with baricitinib or treated with 10 µM filgotinib. However, in the highest concentration of 10 µM, filgotinib among all four JAKi induced the strongest proliferation inhibition in freshly stimulated and in preactivated PBMCs.

Furthermore, behavior of JAKi on lymphocyte proliferation is also reflected by data obtained from CD25 analysis, supporting an antiproliferative rather than a cytotoxic impact of JAKi. Of note, CD25 represents the interleukin-2 receptor α-chain (IL-2Rα) being part of the high-affinity IL-2 receptor [39]. Its surface expression is regulated by initial T cell receptor (TCR) activation and delayed IL-2 receptor stimulation [40]. Since IL-2R signal transduction depends on JAK/STAT pathways, impairment of JAK/STAT signaling inhibited CD25 expression and T cell proliferation [40,41]. Further, it was reported that application of JAK1/2 inhibitor ruxolitinib suppressed IL-2-induced STAT5 phosphorylation and CD25 expression, whereas phosphorylation of molecules associated with early T cell receptor signaling was not affected [42].

Our data obtained from MTX-treated samples (≥0.1 µM) demonstrated reduced CD25 expression, but to a lesser extent compared to JAKi-application, while MTX caused a strong proliferation inhibition of freshly stimulated and preactivated PBMCs. These results are in line with values obtained from 3-(4,5-dimethylthiazol-2-yl)-2,5-diphenyl-2H-tetrazolium bromide (MTT) assay by Nesher et al., stating a significant proliferation inhibition of mitogen-stimulated PBMCs when treated with MTX concentrations > 10 nM for 72 h [43].

To further distinguish proliferation inhibition from cytotoxicity, we additionally performed cell death analysis by Annexin V/PI staining. Treatment of freshly activated PBMCs with a high dose (10 µM) of tofacitinib, upadacitinib, or filgotinib induced a significant increase of apoptotic cells from 36% to 53%. Cell death was also enhanced in preactivated PBMCs treated with high concentrations of JAK1-selective JAKi upadacitinib or filgotinib. Therefore, the decreased proliferation rate after high dose JAKi exposure might at least partly be due to induced cell death. Whereas viability of unstimulated PBMCs treated with MTX was not significantly affected in the applied setting, a strong increase of apoptosis was determined for MTX treatment of freshly and preactivated lymphocytes, leading to the conclusion that this reduced lymphocyte proliferation was mainly due to cytotoxicity. As already shown in publications from the 1990s, MTX primarily targets highly proliferating lymphocytes, mainly in the S phase of the cell cycle, while resting T cells were only little affected [44,45]. Therefore, only low-dose MTX therapy is applied in patients suffering from RA. Nevertheless, also this approach can induce drug toxicity, forcing patients to change the treatment method [46,47,48].

Cell death can be induced by enhanced cytotoxic but also by genotoxic stress, when DNA lesions cannot be repaired efficiently. DNA DSBs are among the most lethal types of DNA damage. A widely used marker to analyze DNA DSBs and DNA repair is γH2AX, a core histone protein rapidly phosphorylated on serin-139 by ataxia telangiectasia mutated protein (ATM), ATM- and Rad3-related (ATR), or DNA-dependent protein kinase (DNA-PK) in chromatin surrounding the break site [25,27]. Immunofluorescence staining followed by quantification of individual γH2AX foci represents the most sensitive method to detect DSBs [25,27,28]. DNA DSBs are repaired by two major pathways—non-homologous end joining (NHEJ) and homologous recombination (HR). Due to its simplicity NHEJ is the preferred pathway throughout the cell cycle, directly ligating two adjacent DNA DSB ends. In contrast, HR provides higher fidelity but requires a homologues DNA template. Thus, HR is primarily activated in the S/G2 phase of the cell cycle [49,50].

There is increasing evidence that DNA damage repair is also modulated by JAK/STAT signaling [20,21,22,23,24]. Further, inhibiting JAK/STAT pathways, e.g., by the JAK1/2 inhibitor ruxolitinib, impaired HR and NHEJ, and was accompanied by reduced expression of different proteins involved in DNA damage response [23]. To analyze the impact of different JAKi on DSB induction, we quantified γH2AX and 53BP1 foci 24 h after JAKi treatment. Additionally, we measured residual γH2AX and 53BP1 foci in unstimulated lymphocytes 24 h after irradiation with 2 Gy in the presence of increasing JAKi concentrations, to investigate their impact on DNA repair. A significant increase of both DSB markers in non-irradiated and irradiated samples was determined in PBMCs after incubation with 10 µM filgotinib. Further, a dose-dependent accumulation of residual γH2AX-foci 24 h after radiation was observed in samples treated with pan-JAKi tofacitinib or baricitinib, whereas upadacitinib and MTX did not lead to enhanced levels of DBS foci.

Microscopic quantification of γH2AX foci represents the most sensitive approach when samples with low γH2AX levels were investigated. In contrast, flow cytometric measurement of intracellular γH2AX intensity is more suitable in samples with high γH2AX expression, where individual foci cannot be distinguished properly. Furthermore, flow cytometry offers the advantage of simultaneous DNA content analysis, since γH2AX levels differ depending on the cell cycle phase. DNA and histone content and intrinsic γH2AX expression increase as cells progressing from G_1_ to S, G_2_, and M phase [51]. Therefore, it is recommended to combine γH2AX quantification of proliferating cells with cell cycle analysis.

In the present study, DSB analysis had to be restricted to resting PBMCs since difficulties were encountered quantifying the γH2AX level in PHA-stimulated lymphocytes. The vast majority of isolated lymphocytes are in a resting state (G_0_) exhibiting only a low baseline γH2AX level. Activation by antigen or mitogen stimulus induces chromatin remodeling and transcriptional activation. Thus, transition of lymphocytes from G_0_ to G_1_ phase also involves a strong endogenous induction of γH2AX [52]. In contrast to cells in S and G_2_/M, G_1_ cells cannot be differentiated from the G_0_ phase by DNA content analysis. Preliminary data of flow cytometric γH2AX measurements combined with cell cycle staining based on DNA content revealed a reduction of γH2AX positive cells with increasing JAKi concentration, while a dose of 10 µM again induced a small rise in γH2AX intensity. Since cell proliferation and thereby high expression of intrinsic γH2AX in activated lymphocytes was inhibited with increasing concentrations of JAKi, this method was not sufficient to distinguish JAKi induced DNA damage or modulated DNA repair from endogenous γH2AX expression, which varied depending on activation status and cell cycle phase. Therefore, we discontinued this analysis of stimulated PBMCs. In future studies, modified protocols need to be established including additional proliferation markers, such as Ki67 [53]. Furthermore, precise differentiation of cells regarding their cell cycle phase will also allow one to analyze the influence of JAKi on proteins critical for HR, such as RAD51, which is primarily active in the S/G_2_ cell cycle phase.

Although preclinical analysis applying multiple standardized genotoxicity assays did not reveal an enhanced DNA damaging potential of approved JAKi, there is increasing evidence that JAK/STAT signaling is involved in DNA damage repair and modulates chemo- and radiosensitivity. As reviewed in detail elsewhere, various in vivo and in vitro data demonstrated hyperactivation of JAK/STAT signaling, especially STAT3, in cancer cells contributing to cancer progression and radio- and chemoresistance [24,54,55,56]. STAT3 activation induced upregulation of DNA repair molecules, while STAT3 deficiency induced downregulation of proteins especially involved in DSB sensing and repair through HR, e.g., RAD51 [20,24,56]. Furthermore, the application of JAK inhibitor ruxolitinib downregulated key proteins of HR and NHEJ, thereby reducing DNA repair activity [22,23]. Bonner et al. also reported enhanced radiosensitivity and reduced DNA DSB repair in irradiated head and neck cancer cells treated with radiosensitizer cetuximab in combination with a JAK1 inhibitor [21]. Furthermore, Maranto et al. reported JAK2/STAT5A/B-dependent expression of Rad51 and suppression of HR but not NHEJ when STAT5A/B was knocked down [57].

Although JAKis show differences in their JAK-isoform selectivity, high dosage can also induce inhibition of additional JAK isoforms and off-target effects [4]. Based on data from pharmacological reviews published by FDA and EMA, mean maximal plasma concentrations (c_max_) in human subjects treated with tofacitinib (5 mg/twice a day), baricitinib (4 mg/day), or upadacitinib (15 mg/day) reach values of approximately 0.15 µM [58,59,60,61,62,63]. A mean maximal plasma concentrations between 4 and 6.1 µM was reported after application of filgotinib (200 mg/day) [64,65]. Regarding published IC_50_ values obtained by cell-free enzymatic assays (summarized in Table S1), reported c_max_ exceed IC_50_ values of multiple JAK isoforms. With respect to doses used in our in vitro study, reported c_max_ of tofacitinib, baricitinib, and upadacitinib are multiple folds below concentrations associated with significant increases of apoptosis or γH2AX foci, whereas published cmax of filgotinib is in a magnitude comparable with the highest concentration (10 µM) applied. As JAKis show distinct pharmacokinetic profiles, e.g., hours per day above IC_50_ values and average daily STAT inhibition among different human leucocyte subpopulations need to be considered to evaluate particular safety and efficacy.

Such concentration-time profiles have been calculated for different JAKi by McInnes et al. [17] and Traves et al. [66]. Investigating cytokine-stimulated STAT activation authors reported similar daily average inhibition of JAK1-dependent signaling pathways. However, Travers et al. described highest JAK-1 selectivity of filgotinib, showing the least inhibition of JAK2- and JAK3-associated pathways when compared with tofacitinib, baricitinib, and upadacitinib and the least inhibition of JAK1/JAK3-related cytokines, such as IL-2, IL-15, or IL-21 [66].

Of note, IL-15 and IL-21 mediate proliferation and function of natural killer (NK) cells, which are essential for clearance of virus-infected and tumorigenic cells. JAKi treatment with tofacitinib and upadacitinib [17,67,68,69,70,71], but not with baricitinib or filgotinib [35,72,73], was accompanied with mild to moderate decrease of circulating NK cell number and impaired NK cell function. Although, these effects were not associated with an increased risk of infectious diseases or lymphoma. Investigating the effect of JAKi ruxolitinib in a murine breast cancer model Bottos et al. showed a JAKi-induced impairment of NK cell-mediated tumor immunosurveillance and enhanced metastasis formation, which were overcome by immunostimulation with IL-15 [74]. Until now, clinical relevance of JAKi-induced NK cell inhibition in RA treatment remains unclear [17,67,69]. Though, especially in regard with potentially affected DNA damage repair, this aspect also needs to be further investigated in long-term studies.

Cancer cells often exhibit dysregulations in DNA repair mechanisms. However, several studies also indicated enhanced DNA damage and DNA repair deficiencies in lymphocytes from RA patients [75,76,77,78]. In addition to studies investigating the role of different JAK inhibitors on DNA damage response pathways in primary cells from healthy donors or cancer cell lines, future trials also need to address the impact of JAKi on lymphocytes from patients with RA.

## 5. Conclusions

In conclusion, our study confirmed a comparable, dose-dependent inhibition of lymphocyte activation and proliferation in PBMCs treated with tofacitinib, baricitinib, and upadacitinib, independent of JAK selectivity. In line with reported IC_50_ values regarding JAK1-enzymatic activity and administered dosage in vivo concentrations of filgotinib had to be approximately two orders of magnitude higher to induce significant immunomodulatory effects. Furthermore, antiproliferating properties especially of JAK1-selective inhibitors may at least partially be caused by cytotoxicity, since high doses also affected cell viability. For the first time the effect of tofacitinib, baricitinib, upadacitinib, and filgotinib on DNA DSB induction and repair of radiation-induced DNA damage was investigated by applying γH2AX and 53BP1 foci analysis. Our results provide first evidence for a significant increase in DNA DSB markers after exposure to 10 µM filgotinib and a dose-dependent enrichment of residual γH2AX foci in irradiated samples incubated with pan-JAKi tofacitinib and baricitinib, possibly indicating attenuated DNA damage repair. Although these in vitro results do not necessarily represent behavior in vivo, additional studies need to be performed further investigating the impact of approved JAK inhibition on DNA damage response and their potential long-term effects in vitro and in vivo, also comprising analysis of RA patients. Since JAKi are also administered in combination with MTX, the impact of combined treatment additionally needs to be addressed in future trials, especially as MTX itself demonstrated JAK/STAT pathway inhibiting properties [79,80].

## Supplementary Materials

The following are available online at https://www.mdpi.com/article/10.3390/jcm10071431/s1, Table S1: Summary of mean half-maximal inhibitory concentrations (IC_50_) obtained by enzymatic assays with indicated ATP concentration (c_ATP_) for different JAKi, Figure S1: Proliferation analysis by ^3^H-thymidine incorporation assay 72 h after PHA-stimulation of PBMCs treated with indicated concentrations of methotrexate (MTX) either (a) immediately after activation or (b) 48 h after PHA-stimulation.

## References

[1] Smolen J.S., Aletaha D., Barton A., Burmester G.R., Emery P., Firestein G.S., Kavanaugh A., McInnes I.B., Solomon D.H., Strand V. (2018). Rheumatoid arthritis. Nat. Rev. Dis. Prim..

[2] Semerano L., Decker P., Clavel G., Boissier M.C. (2016). Developments with investigational Janus kinase inhibitors for rheumatoid arthritis. Expert Opin. Investig. Drugs.

[3] Burmester G.R., Bijlsma J.W.J., Cutolo M., McInnes I.B. (2017). Managing rheumatic and musculoskeletal diseases-past, present and future. Nat. Rev. Rheumatol..

[4] Bechman K., Yates M., Galloway J.B. (2019). The new entries in the therapeutic armamentarium: The small molecule JAK inhibitors. Pharmacol. Res..

[5] Krüger K. (2019). Role of janus kinase inhibitors in the treatment of rheumatic diseases. Internist.

[6] Winthrop K.L. (2017). The emerging safety profile of JAK inhibitors in rheumatic disease. Nat. Rev. Rheumatol..

[7] Damsky W., Peterson D., Ramseier J., Al-Bawardy B., Chun H., Proctor D., Strand V., Flavell R.A., King B. (2020). The emerging role of Janus kinase inhibitors in the treatment of autoimmune and inflammatory diseases. J. Allergy Clin. Immunol..

[8] Menet C.J., Van Rompaey L., Geney R. (2013). Advances in the Discovery of Selective JAK Inhibitors. Prog. Med. Chem..

[9] Salas A., Hernandez-Rocha C., Duijvestein M., Faubion W., McGovern D., Vermeire S., Vetrano S., Vande Casteele N. (2020). JAK–STAT pathway targeting for the treatment of inflammatory bowel disease. Nat. Rev. Gastroenterol. Hepatol..

[10] Schwartz D.M., Kanno Y., Villarino A., Ward M., Gadina M., O’Shea J.J. (2017). JAK inhibition as a therapeutic strategy for immune and inflammatory diseases. Nat. Rev. Drug Discov..

[11] O’Shea J.J., Plenge R. (2013). JAKs and STATs in Immunoregulation and Immune-Mediated Disease. Immunity.

[12] Villarino A.V., Kanno Y., O’Shea J.J. (2017). Mechanisms and consequences of Jak-STAT signaling in the immune system. Nat. Immunol..

[13] Harrington R., Al Nokhatha S.A., Conway R. (2020). Jak inhibitors in rheumatoid arthritis: An evidence-based review on the emerging clinical data. J. Inflamm. Res..

[14] Dhillon S., Keam S.J. (2020). Filgotinib: First Approval. Drugs.

[15] O’Shea J.J., Gadina M. (2019). Selective Janus kinase inhibitors come of age. Nat. Rev. Rheumatol..

[16] Choy E.H. (2019). Clinical significance of Janus Kinase inhibitor selectivity. Rheumatology.

[17] McInnes I.B., Byers N.L., Higgs R.E., Lee J., Macias W.L., Na S., Ortmann R.A., Rocha G., Rooney T.P., Wehrman T. (2019). Comparison of baricitinib, upadacitinib, and tofacitinib mediated regulation of cytokine signaling in human leukocyte subpopulations. Arthritis Res. Ther..

[18] Russell A.S. (1990). Activated lymphocytes in the peripheral blood of patients with rheumatoid arthritis. J. Rheumatol..

[19] Kitanaga Y., Imamura E., Nakahara Y., Fukahori H., Fujii Y., Kubo S., Nakayamada S., Tanaka Y. (2020). In vitro pharmacological effects of peficitinib on lymphocyte activation: A potential treatment for systemic sclerosis with JAK inhibitors. Rheumatology.

[20] Barry S.P., Townsend P.A., Knight R.A., Scarabelli T.M., Latchman D.S., Stephanou A. (2010). STAT3 modulates the DNA damage response pathway. Int. J. Exp. Pathol..

[21] Bonner J.A., Trummell H.Q., Bonner A.B., Willey C.D., Bredel M., Yang E.S. (2015). Enhancement of Cetuximab-Induced Radiosensitization by JAK-1 Inhibition. BMC Cancer.

[22] Gupta P., Sharma Y., Viswanathan P., Gupta S. (2020). Cellular cytokine receptor signaling and ATM pathway intersections affect hepatic DNA repair. Cytokine.

[23] Nieborowska-Skorska M., Maifrede S., Dasgupta Y., Sullivan K., Flis S., Le B.V., Solecka M., Belyaeva E.A., Kubovcakova L., Nawrocki M. (2017). Ruxolitinib-induced defects in DNA repair cause sensitivity to PARP inhibitors in myeloproliferative neoplasms. Blood.

[24] Yang P.L., Liu L.X., Li E.M., Xu L.Y. (2020). Stat3, the challenge for chemotherapeutic and radiotherapeutic efficacy. Cancers.

[25] Bonner W.M., Redon C.E., Dickey J.S., Nakamura A.J., Sedelnikova O.A., Solier S., Pommier Y. (2008). GammaH2AX and cancer. Nat. Rev. Cancer.

[26] Marková E., Schultz N., Belyaev I.Y. (2007). Kinetics and dose-response of residual 53BP1/γ-H2AX foci: Co-localization, relationship with DSB repair and clonogenic survival. Int. J. Radiat. Biol..

[27] Rothkamm K., Barnard S., Moquet J., Ellender M., Rana Z., Burdak-Rothkamm S. (2015). DNA damage foci: Meaning and significance. Environ. Mol. Mutagen..

[28] Reddig A., Roggenbuck D., Reinhold D. (2018). Comparison of different immunoassays for γH2AX quantification. J. Lab. Precis. Med..

[29] Willitzki A., Lorenz S., Hiemann R., Guttek K., Goihl A., Hartig R., Conrad K., Feist E., Sack U., Schierack P. (2013). Fully automated analysis of chemically induced γH2AX foci in human peripheral blood mononuclear cells by indirect immunofluorescence. Cytom. A.

[30] Wöltgens J.H.M., Lyaruu D.M., Bronckers A.L.J.J., Van Duin M.A., Bervoets T.J.M. (1998). Effect of methotrexate on cell proliferation in developing hamster molar tooth germs in vitro. Eur. J. Oral Sci..

[31] Lefevre P.L.C., Vande Casteele N. (2020). Clinical Pharmacology of Janus Kinase Inhibitors in Inflammatory Bowel Disease. J. Crohns. Colitis.

[32] Angelini J., Talotta R., Roncato R., Fornasier G., Barbiero G., Cin L.D., Brancati S., Scaglione F. (2020). JAK-inhibitors for the treatment of rheumatoid arthritis: A focus on the present and an outlook on the future. Biomolecules.

[33] Gilead Sciences Gilead Receives Complete Response Letter for Filgotinib for the Treatment of Moderately to Severely Active Rheumatoid Arthritis. https://www.businesswire.com/news/home/20200818005811/en/.

[34] Markham A., Keam S.J. (2019). Peficitinib: First Global Approval. Drugs.

[35] Tanaka Y., Izutsu H. (2020). Peficitinib for the treatment of rheumatoid arthritis: An overview from clinical trials. Expert Opin. Pharmacother..

[36] Pope J., Sawant R., Tundia N., Du E.X., Qi C.Z., Song Y., Tang P., Betts K.A. (2020). Comparative Efficacy of JAK Inhibitors for Moderate-To-Severe Rheumatoid Arthritis: A Network Meta-Analysis. Adv. Ther..

[37] Lee Y.H., Song G.G. (2020). Comparative efficacy and safety of tofacitinib, baricitinib, upadacitinib, and filgotinib in active rheumatoid arthritis refractory to biologic disease-modifying antirheumatic drugs. Z. Rheumatol..

[38] Dowty M.E., Lin T.H., Jesson M.I., Hegen M., Martin D.A., Katkade V., Menon S., Telliez J.B. (2019). Janus kinase inhibitors for the treatment of rheumatoid arthritis demonstrate similar profiles of in vitro cytokine receptor inhibition. Pharmacol. Res. Perspect..

[39] Damoiseaux J. (2020). The IL-2—IL-2 receptor pathway in health and disease: The role of the soluble IL-2 receptor. Clin. Immunol..

[40] Shatrova A.N., Mityushova E.V., Vassilieva I.O., Aksenov N.D., Zenin V.V., Nikolsky N.N., Marakhova I.I. (2016). Time-dependent regulation of IL-2R α-chain (CD25) expression by TCR signal strength and IL-2-induced STAT5 signaling in activated human blood T lymphocytes. PLoS ONE.

[41] Nakajima H., Liu X.W., Wynshaw-Boris A., Rosenthal L.A., Imada K., Finbloom D.S., Hennighausen L., Leonard W.J. (1997). An indirect effect of Stat5a in IL-2-induced proliferation: A critical role for stat5a in IL-2-mediated IL-2 receptor α chain induction. Immunity.

[42] Parampalli Yajnanarayana S., Stübig T., Cornez I., Alchalby H., Schönberg K., Rudolph J., Triviai I., Wolschke C., Heine A., Brossart P. (2015). JAK1/2 inhibition impairs T cell function in vitro and in patients with myeloproliferative neoplasms. Br. J. Haematol..

[43] Nesher G., Moore T.L. (1990). The in vitro effects of methotrexate on peripheral blood mononuclear cells: Modulation by methyl donors and spermidine. Arthritis Rheum..

[44] Genestier L., Paillot R., Fournel S., Ferraro C., Miossec P., Revillard J.P. (1998). Immunosuppressive properties of methotrexate: Apoptosis and clonal deletion of activated peripheral T cells. J. Clin. Investig..

[45] Fairbanks L.D., Rückemann K., Qiu Y., Hawrylowicz C.M., Richards D.F., Swaminathan R., Kirschbaum B., Simmonds H.A. (1999). Methotrexate inhibits the first committed step of purine biosynthesis in mitogen-stimulated human T-lymphocytes: A metabolic basis for efficacy in rheumatoid arthritis?. Biochem. J..

[46] Herman S., Zurgil N., Deutsch M. (2005). Low dose methotrexate induces apoptosis with reactive oxygen species involvement in T lymphocytic cell lines to a greater extent than in monocytic lines. Inflamm. Res..

[47] Romão V.C., Lima A., Bernardes M., Canhão H., Fonseca J.E. (2014). Three decades of low-dose methotrexate in rheumatoid arthritis: Can we predict toxicity?. Immunol. Res..

[48] Bedoui Y., Guillot X., Sélambarom J., Guiraud P., Giry C., Jaffar-Bandjee M.C., Ralandison S., Gasque P. (2019). Methotrexate an old drug with new tricks. Int. J. Mol. Sci..

[49] Vítor A.C., Huertas P., Legube G., de Almeida S.F. (2020). Studying DNA Double-Strand Break Repair: An Ever-Growing Toolbox. Front. Mol. Biosci..

[50] Jeggo P.A., Löbrich M. (2015). How cancer cells hijack DNA double-strand break repair pathways to gain genomic instability. Biochem. J..

[51] Turinetto V., Giachino C. (2015). Survey and summary multiple facets of histone variant H2AX: A DNA double-strand-break marker with several biological functions. Nucleic Acids Res..

[52] Tanaka T., Kajstura M., Halicka H.D., Traganos F., Darzynkiewicz Z. (2007). Constitutive histone H2AX phosphorylation and ATM activation are strongly amplified during mitogenic stimulation of lymphocytes. Cell Prolif..

[53] Vignon C., Debeissat C., Georget M.T., Bouscary D., Gyan E., Rosset P., Herault O. (2013). Flow Cytometric Quantification of All Phases of the Cell Cycle and Apoptosis in a Two-Color Fluorescence Plot. PLoS ONE.

[54] Hall W.A., Sabharwal L., Udhane V., Maranto C., Nevalainen M.T. (2020). Cytokines, JAK-STAT Signaling and Radiation-Induced DNA Repair in Solid Tumors: Novel Opportunities for Radiation Therapy. Int. J. Biochem. Cell Biol..

[55] Spitzner M., Ebner R., Wolff H.A., Michael Ghadimi B., Wienands J., Grade M. (2014). STAT3: A novel molecular mediator of resistance to chemoradiotherapy. Cancers.

[56] Wang X., Zhang X., Qiu C., Yang N. (2020). STAT3 Contributes to Radioresistance in Cancer. Front. Oncol..

[57] Maranto C., Udhane V., Hoang D.T., Gu L., Alexeev V., Malas K., Cardenas K., Brody J.R., Rodeck U., Bergom C. (2018). STAT5A/B Blockade Sensitizes Prostate Cancer to Radiation through Inhibition of RAD51 and DNA Repair. Clin. Cancer Res..

[58] Pharmacology Review NDAReference ID: 3205502. https://www.accessdata.fda.gov/drugsatfda_docs/nda/2012/203214Orig1s000PharmR.pdf.

[59] Pharmacology Review NDAReference ID: 4261989. https://www.accessdata.fda.gov/drugsatfda_docs/nda/2018/207924Orig1s000PharmR.pdf.

[60] Clinical Pharmacology and Biopharmaceutics Review NDAReference ID: 4435111. https://www.accessdata.fda.gov/drugsatfda_docs/nda/2019/211675Orig1s000ClinPharmR.pdf.

[61] Assessment Report EMA/CHMP/853224/2016. https://www.ema.europa.eu/en/documents/assessment-report/xeljanz-epar-public-assessment-report_en.pdf.

[62] Assessment Report EMA/520470/2020. https://www.ema.europa.eu/en/documents/variation-report/olumiant-h-c-4085-ii-0016-epar-assessment-report-variation_en.pdf.

[63] Assessment Report EMA/608624/2019. https://www.ema.europa.eu/en/documents/assessment-report/rinvoq-epar-public-assessment-report_en.pdf.

[64] Assessment Report EMA/424374/2020. https://www.ema.europa.eu/en/documents/assessment-report/jyseleca-epar-public-assessment-report_en.pdf.

[65] Vanhoutte F., Mazur M., Voloshyn O., Stanislavchuk M., Van der Aa A., Namour F., Galien R., Meuleners L., van ’t Klooster G. (2017). Efficacy, Safety, Pharmacokinetics, and Pharmacodynamics of Filgotinib, a Selective JAK-1 Inhibitor, After Short-Term Treatment of Rheumatoid Arthritis: Results of Two Randomized Phase IIa Trials. Arthritis Rheumatol..

[66] Traves P.G., Murray B., Campigotto F., Galien R., Meng A., Di Paolo J.A. (2021). JAK selectivity and the implications for clinical inhibition of pharmacodynamic cytokine signalling by filgotinib, upadacitinib, tofacitinib and baricitinib. Ann. Rheum. Dis..

[67] Kameda H., Takeuchi T., Yamaoka K., Oribe M., Kawano M., Zhou Y., Othman A.A., Pangan A.L., Kitamura S., Meerwein S. (2020). Efficacy and safety of upadacitinib in Japanese patients with rheumatoid arthritis (SELECT-SUNRISE): A placebo-controlled phase IIb/III study. Rheumatology.

[68] Weinhold K.J., Bukowski J.F., Brennan T.V., Noveck R.J., Staats J.S., Lin L., Stempora L., Hammond C., Wouters A., Mojcik C.F. (2018). Reversibility of peripheral blood leukocyte phenotypic and functional changes after exposure to and withdrawal from tofacitinib, a Janus kinase inhibitor, in healthy volunteers. Clin. Immunol..

[69] Nocturne G., Pascaud J., Ly B., Tahmasebi F., Mariette X. (2020). JAK inhibitors alter NK cell functions and may impair immunosurveillance against lymphomagenesis. Cell. Mol. Immunol..

[70] Parmentier J.M., Voss J., Graff C., Schwartz A., Argiriadi M., Friedman M., Camp H.S., Padley R.J., George J.S., Hyland D. (2018). In vitro and in vivo characterization of the JAK1 selectivity of upadacitinib (ABT-494). BMC Rheumatol..

[71] Genovese M.C., Smolen J.S., Weinblatt M.E., Burmester G.R., Meerwein S., Camp H.S., Wang L., Othman A.A., Khan N., Pangan A.L. (2016). Efficacy and Safety of ABT-494, a Selective JAK-1 Inhibitor, in a Phase IIb Study in Patients With Rheumatoid Arthritis and an Inadequate Response to Methotrexate. Arthritis Rheumatol..

[72] Tarrant J.M., Galien R., Li W., Goyal L., Pan Y., Hawtin R., Zhang W., Van der Aa A., Taylor P.C. (2020). Filgotinib, a JAK1 Inhibitor, Modulates Disease-Related Biomarkers in Rheumatoid Arthritis: Results from Two Randomized, Controlled Phase 2b Trials. Rheumatol. Ther..

[73] Galien R., Brys R., Van Der Aa A., Harrison P., Tasset C. (2015). Absence of effects of filgotinib on erythrocytes, CD8+ and NK cells in rheumatoid arthritis patients brings further evidence for the JAK1 selectivity of filgotinib. Arthritis Rheumatol..

[74] Bottos A., Gotthardt D., Gill J.W., Gattelli A., Frei A., Tzankov A., Sexl V., Wodnar-Filipowicz A., Hynes N.E. (2016). Decreased NK-cell tumour immunosurveillance consequent to JAK inhibition enhances metastasis in breast cancer models. Nat. Commun..

[75] Galita G., Brzezińska O., Gulbas I., Sarnik J., Poplawska M., Makowska J., Poplawski T. (2020). Increased Sensitivity of PBMCs Isolated from Patients with Rheumatoid Arthritis to DNA Damaging Agents Is Connected with Inefficient DNA Repair. J. Clin. Med..

[76] Souliotis V.L., Vlachogiannis N.I., Pappa M., Argyriou A., Sfikakis P.P. (2019). DNA damage accumulation, defective chromatin organization and deficient DNA repair capacity in patients with rheumatoid arthritis. Clin. Immunol..

[77] Shao L. (2018). Dna damage response signals transduce stress from rheumatoid arthritis risk factors into t cell dysfunction. Front. Immunol..

[78] Li Y., Goronzy J.J., Weyand C.M. (2018). DNA damage, metabolism and aging in pro-inflammatory T cells: Rheumatoid arthritis as a model system. Exp. Gerontol..

[79] Gremese E., Alivernini S., Tolusso B., Zeidler M.P., Ferraccioli G. (2019). JAK inhibition by methotrexate (and csDMARDs) may explain clinical efficacy as monotherapy and combination therapy. J. Leukoc. Biol..

[80] Thomas S., Fisher K.H., Snowden J.A., Danson S.J., Brown S.P. (2015). Methotrexate is a JAK/STAT pathway inhibitor. PLoS ONE.

