# Supplementary material for: Impact of Different JAK Inhibitors and Methotrexate on Lymphocyte Proliferation and DNA Damage
Source: J Clin Med. 2021 Apr 1;10(7):1431. doi: 10.3390/jcm10071431 (PMC8036268; doi:10.3390/jcm10071431)
Supplement: Supplementary file 1 [file jcm-10-01431-s001.pdf]

Journal of Clinical Medicine

Table S1. Summary of mean half-maximal inhibitory concentrations (IC_{50}) obtained by enzymatic assays with indicated ATP concentration (c_{ATP}) for different JAKi.

JAKi	IC50 [nM]				c_{ATP}	Reference
	JAK1	JAK2	JAK3	TYK2	[mM]	
Tofacitinib	15	71	45	472	1	[1]
	15.1	77.4	55	489	1	[2]
Baricitinib	0.78	2	253	14	1	[1]
	4	6.6	787	61	1	[2]
	5.9	5	>400	53	1	[3]
Upadacitinib	0.76	19	224	118	1	[1]
	47	120	2304	4690	0.1	[4]
	43	120	2300	4700	0.1	[5]
Filgotinib	45	357	9097	397	1	[1]
	363	2400	>10000	2600	1	[2]
	10-53	28-29	311-810	116-177	0.01	[6]

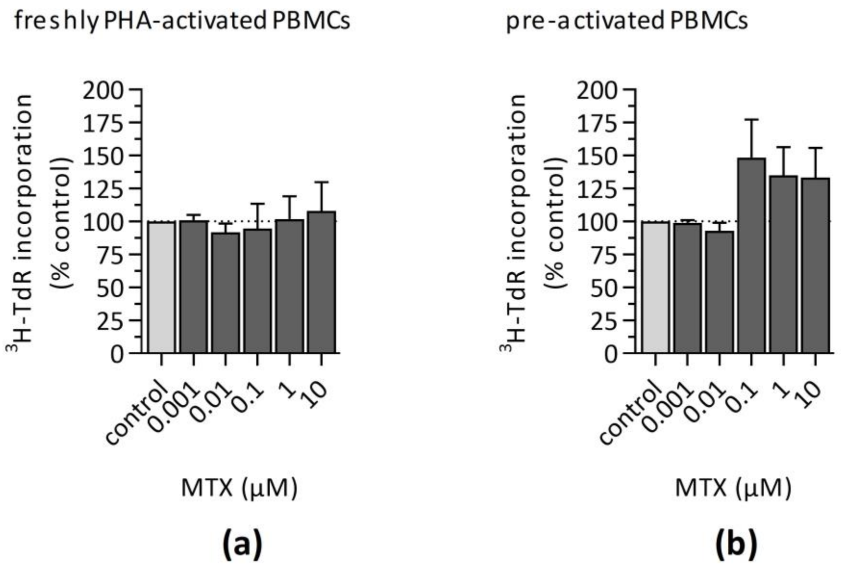

Figure 1. Proliferation analysis by ^{3}H-thymidine incorporation assay 72 h after PHA-stimulation of PBMCs treated with indicated concentrations of methotrexate (MTX) either (**a**) immediately after activation or (**b**) 48 h after PHA-stimulation. Diagrams display the mean ± SEM of five independent experiments normalized to DMSO control (*** $p \leq 0.001$; ** $p \leq 0.01$; * $p \leq 0.05$). As described in the result section ^{3}H-thymidine incorporation assay was not suitable to study MTX-induced proliferation inhibition since MTX effects internal thymidine biosynthesis.

References

1. Dowty, M.E.; Lin, T.H.; Jesson, M.I.; Hegen, M.; Martin, D.A.; Katkade, V.; Menon, S.; Telliez, J.B. Janus kinase inhibitors for the treatment of rheumatoid arthritis demonstrate similar profiles of in vitro cytokine receptor inhibition. *Pharmacol. Res. Perspect.* **2019**, *7*, 1–11, doi:10.1002/prp2.537.
2. Clark, J.D.; Flanagan, M.E.; Telliez, J.B. Discovery and development of Janus kinase (JAK) inhibitors for inflammatory diseases. *J. Med. Chem.* **2014**, *57*, 5023–5038, doi:10.1021/jm401490p.

3. Fridman, J.S.; Scherle, P.A.; Collins, R.; Burn, T.C.; Li, Y.; Li, J.; Covington, M.B.; Thomas, B.; Collier, P.; Favata, M.F.; et al. Selective Inhibition of JAK1 and JAK2 Is Efficacious in Rodent Models of Arthritis: Preclinical Characterization of INCB. *J. Immunol.* **2010**, *184*, 5298–5307, doi:10.4049/jimmunol.0902819.
4. Parmentier, J.M.; Voss, J.; Graff, C.; Schwartz, A.; Argiriadi, M.; Friedman, M.; Camp, H.S.; Padley, R.J.; George, J.S.; Hyland, D.; et al. In vitro and in vivo characterization of the JAK1 selectivity of upadacitinib (ABT-494). *BMC Rheumatol.* **2018**, *2*, 1–11, doi:10.1186/s41927-018-0031-x.
5. Assessment Report EMA/608624/2019. Available online: https://www.ema.europa.eu/en/documents/assessment-report/rinvoq-epar-public-assessment-report_en.pdf (accessed on 18 March 2021).
6. Assessment Report EMA/424374/2020. Available online: https://www.ema.europa.eu/en/documents/assessment-report/jyseleca-epar-public-assessment-report_en.pdf (accessed on 18 March 2021).
